# Superior shoulder suspensory complex disruptions: from injury pattern recognition to treatment decision-making—a narrative review with institutional case illustrations

**DOI:** 10.1016/j.xrrt.2026.100793

**Published:** 2026-06-12

**Authors:** Muhammed Yusuf Afacan, Doruk Akgün, Elmar-Constantin Kuehnle, Jean-Baptiste Quast-Cojocaru, Soraya Bahlawane, Yusuf Inanc, Agahan Hayta, Rony-Orijit Dey Hazra, David Alexander Back, Alp Paksoy

**Affiliations:** a Istanbul Physical Therapy and Rehabilitation Training and Research Hospital, Department of Orthopaedics and Traumatology, Istanbul, Turkiye; b Istanbul University-Cerrahpasa, Institute of Graduate Studies, Cerrahpasa Faculty of Medicine, Department of Anatomy, Istanbul, Turkiye; c Charité-Universitätsmedizin Berlin, Center for Musculoskeletal Surgery, Corporate Member of Freie Universität Berlin and Humboldt-Universität zu Berlin, Berlin, Germany

**Keywords:** Superior shoulder suspensory complex, Floating shoulder, Scapular fracture, Acromioclavicular joint, Coracoid fracture, Shoulder girdle trauma, omputed tomography, Treatment algorithm

## Abstract

**Background:**

Disruptions of the superior shoulder suspensory complex (SSSC) represent an uncommon and heterogeneous spectrum of shoulder girdle injuries. Because the available literature is largely limited to case reports, small case series, and technical descriptions, management decisions remain challenging, particularly for double, triple, and quadruple disruption patterns. A clinically oriented synthesis is needed to translate fragmented evidence into practical diagnostic and treatment guidance. The purpose of this narrative review was to synthesize the available literature on SSSC disruptions into a practical diagnostic and treatment framework, supplemented by institutional illustrative cases. Emphasis was placed on injury pattern recognition, stepwise imaging assessment, instability markers, operative decision-making, and patient-specific modifiers, including age, bone quality, comorbidities, and functional demand.

**Methods:**

A narrative review of the literature was performed, including original articles, case reports, case series, technical notes, review articles, and book chapters addressing single, double, triple, and quadruple SSSC disruptions. Evidence was synthesized according to clinically relevant domains, including mechanism of injury, number of disrupted sites, radiographic and computed tomography (CT) findings, instability markers, operative versus nonoperative indications, fixation principles, and reported outcomes. Institutional cases were included as illustrative examples of the proposed framework rather than as a comparative outcome cohort.

**Results:**

Single SSSC disruptions generally preserve ring stability and are commonly suitable for nonoperative management, unless substantial displacement, intra-articular extension, glenoid or scapular neck involvement, or mechanical compromise is present. Double disruptions require careful assessment of ring instability; fixation of one or more dominant unstable components should be considered when there is significant displacement, reduced glenopolar alignment, medial or lateral translation, angulation, persistent acromioclavicular/coracoclavicular instability, or functional instability. Triple and quadruple disruptions are rare and traditionally associated with high-energy trauma and concomitant injuries; however, complex disruption patterns may also occur after low-energy trauma in elderly or osteoporotic patients. CT, particularly with multiplanar and 3-dimensional reconstruction, is central for defining bony injury patterns, instability markers, and surgical planning, whereas magnetic resonance imaging has a selective role in suspected ligamentous injury, occult fracture, or associated soft tissue pathology.

**Conclusion:**

SSSC disruptions should be approached as ring injuries rather than as isolated fractures or ligamentous lesions. The practical contribution of this review is a structured diagnostic and treatment algorithm that integrates disruption number, radiographic and CT-based instability markers, selective magnetic resonance imaging, patient physiology, bone quality, functional demand, and selective restoration of stability. This framework may help clinicians avoid missed instability, delayed diagnosis, and undertreatment of complex shoulder girdle injuries.

Scapular fractures, constituting less than 1% of all fractures and 3%–5% of shoulder girdle fractures, have a disproportionately significant impact on shoulder stability.^6^^,^^9^ The shoulder girdle consists of 2 interconnected arches: a superior arch, which includes the acromioclavicular (AC) joint (ACJ), clavicle, sternoclavicular (SC) joint, and costoclavicular junction, and an inferior arch, formed by the glenoid cavity, scapula, and scapulothoracic space.^26^ These arches are stabilized by the superior shoulder suspensory complex (SSSC) (Fig. 1), an osseoligamentous ring described firstly by Goss in 1993,^21^ that stabilizes the shoulder girdle by linking the axial skeleton to the upper limb, comprising the glenoid, coracoid process, coracoclavicular (CC) ligament, distal end of the clavicle, ACJ, coracoacromial ligament and acromion.^21^^,^^26^^,^^28^^,^^38^

**Figure 1:**
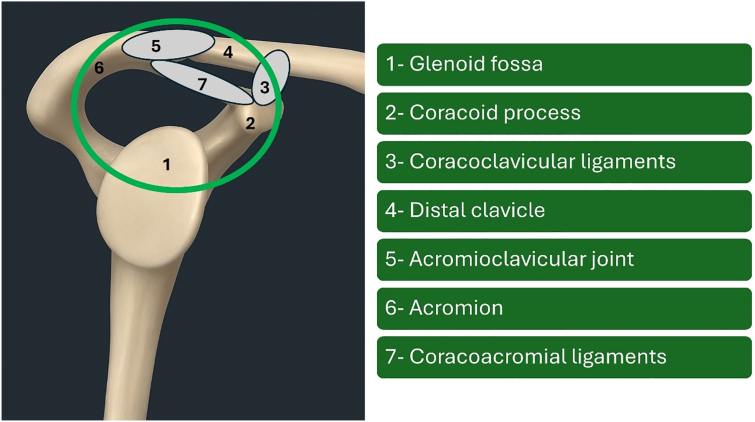
SSSC ring. SSSC is composed of bony elements, including the superior portion of the glenoid (1), the coracoid process (2), distal clavicle (4), acromion (6), and interconnected by the CC ligaments (3), ACJ (5), and CA (7) ligaments. *SSSC*, superior shoulder suspensory complex; CC, coracoclavicular; AC, acromioclavicular; CA, coracoacromial. The biomechanical importance of the SSSC lies in its ring-like configuration. A single disruption may be compensated by the remaining osseoligamentous structures, whereas multiple disruptions can compromise ring continuity, alter scapular position, and destabilize the glenohumeral platform. Therefore, clinically relevant instability depends not only on the injured structure itself but also on whether the superior or inferior strut and the remaining ligamentous stabilizers remain functionally intact.^21^^,^^31^

Segmental injury patterns involving the shoulder are often referred to as a “floating shoulder” or “double disruptions” of the SSSC, as they typically include 2 distinct skeletal and/or ligamentous injuries identifiable on radiographic imaging.^31^ Ganz and Noesberger first introduced the term “floating shoulder” in 1975, defining it as the simultaneous occurrence of ipsilateral fractures of the clavicle and glenoid neck.^17^ Disruptions of the SSSC were afterward defined in the literature as single, double, triple, or quadruple.^21^^,^^28^^,^^36^ Traumatic Rockwood type-2 ACJ sprain and Neer type-1 distal clavicle fractures were counted as a single disruption of the ring.^21^ Goss proposed that a dual disruption involving any 2 of the 4 key structures of the SSSC could result in a “floating” glenohumeral joint.^21^ Triple disruption of the SSSC caused by the breakage of the ring in 3 different locations is an extremely rare injury caused by high-energy trauma.^5^^,^^21^^,^^28^^,^^38^ Quadruple disruption of the SSSC, defined as the breakage of the ring in 4 different locations, is exceedingly rare, with Mulawka et al^36^ reporting only a handful of cases in the literature.

The purpose of this narrative review is not to provide another descriptive catalog of SSSC injuries but to convert the fragmented evidence from case reports, small series, and contemporary imaging studies into a practical decision-making framework. Specifically, we aimed to: (1) clarify the instability implications of single, double, triple, and quadruple disruptions; (2) define a systematic imaging approach based on radiographs, computed tomography (CT), and selective magnetic resonance imaging (MRI); (3) summarize treatment principles according to disruption pattern, displacement, patient physiology, bone quality, and functional demand; and (4) illustrate these principles using institutional cases that represent single, double, and triple disruption patterns.

## Literature search and synthesis strategy

This narrative clinical review was supplemented by institutional illustrative cases and aimed to synthesize fragmented evidence on SSSC disruptions into a practical diagnostic and treatment framework rather than perform a formal systematic review or meta-analysis. A targeted literature search was conducted to identify original articles, case reports, case series, technical notes, reviews, and book chapters addressing single-double-triple-and quadruple SSSC disruptions, floating shoulder injuries, combined clavicle–scapular fractures, AC/CC ligament injuries, and complex shoulder girdle ring injuries. Relevant studies were identified using terms related to SSSC disruption, floating shoulder, scapular neck, glenoid, coracoid, acromion, distal clavicle fractures, ACJ dislocation, and shoulder girdle ring injury, with additional screening of reference lists. Because the evidence mainly consists of case reports, small series, and expert-based descriptions, quantitative pooling was not appropriate. Therefore, studies were synthesized according to mechanism, disruption number, imaging findings, instability markers, treatment indications, fixation principles, complications, and outcomes. Institutional cases were included only as illustrative examples of the proposed framework, with anonymized material and appropriate consent for publication. The epidemiologic and mechanistic characteristics of SSSC disruptions are summarized in Table I.

**Table I:** Epidemiologic and mechanistic characteristics of SSSC disruptions.

Domain	Key findings	Clinical relevance
Frequency according to disruption number	Single SSSC disruptions are more common than multiple disruptions, whereas injury rarity increases as the number of disrupted ring components increases.^28^^,^^36^^,^^41^ Triple and quadruple disruptions are exceedingly rare and are mainly described in case reports or small series.^5^^,^^26^^,^^36^	The number of disrupted sites provides an initial estimate of injury rarity and potential instability.
Double-disruption patterns	Double disruptions may involve 2 fractures, 2 ligamentous injuries, or a combined fracture–ligament injury.^48^^,^^54^	These injuries may create clinically relevant instability when the coracoid, acromion, clavicle, scapular neck, or AC/CC ligament complex is involved.
High-energy mechanisms	Multiple SSSC disruptions are classically associated with motor vehicle collisions, crush injuries, falls from height, direct lateral shoulder impact, and high-energy sports trauma.^1^^,^^5^^,^^28^^,^^36^^,^^48^	These mechanisms should raise suspicion for complex shoulder girdle ring injury.
Associated injuries	Multiple disruptions may coexist with thoracic, spinal, neurologic, ipsilateral upper-extremity, or chest wall injuries.^5^^,^^10^^,^^36^	Associated injuries may influence the timing of definitive shoulder reconstruction and the rehabilitation strategy.^5^^,^^10^^,^^36^
Biomechanical mechanism	The mechanism is often related to lateral-to-medial force transmission across the shoulder girdle or indirect humeral head impaction into the glenoid and scapular neck.^1^^,^^6^^,^^26^	Understanding the force vector helps identify hidden injury components and interpret combined fracture patterns.
Coracoid fracture relevance	Coracoid fractures are uncommon, representing approximately 2%–5% of scapular fractures.^39^^,^^40^	Despite their rarity, coracoid fractures are clinically important because the coracoid contributes to the coracoacromial arch and the clavicle–scapula linkage through the CC and CA ligament complexes.^39^^,^^40^
Coracoid as part of SSSC instability	Coracoid involvement may convert an apparently isolated injury into a mechanically relevant SSSC disruption.	This is particularly important when coracoid fracture is associated with AC/CC instability or acromial, clavicular, or scapular fractures.
Low-energy and elderly/osteoporotic patterns	Institutional illustrative cases suggest that SSSC disruption patterns may not be restricted to the traditional high-energy trauma model. Elderly or osteoporotic patients may sustain complex disruption patterns after relatively low-energy mechanisms.	Although this observation cannot establish incidence or comparative risk, it supports a lower threshold for CT-based evaluation when clinical symptoms exceed plain radiographic findings, especially in older patients whose initial radiographs may underestimate the true extent of shoulder girdle injury.

*AC*, acromioclavicular; *CA*, coracoacromial; *CC*, coracoclavicular; *CT*, computed tomography; *SSSC*, superior shoulder suspensory complex.

## Diagnosis-imaging

In radiographic evaluation of the SSSC injury, the first-choice imaging includes plain X-rays with standard anteroposterior, scapular-Y, and outlet views of the shoulder.^22^ For assessing AC injuries, the Zanca view and for SC joint the Serendipity view are also recommended in some cases.^7^^,^^23^ Panoramic clavicula or shoulder views with and without weight are important to compare the AC and SC joints with the healthy side.^42^ X-rays may effectively detect SSSC injuries such as fractures of the clavicle, scapula (including the coracoid and acromion), and rib, as well as AC and SC joint dislocations, but may require advanced imaging for soft tissue or complex injuries.

CT and MRI play critical roles in the detailed evaluation of SSSC injuries when plain X-rays are insufficient. CT is primarily used for assessing complex bony injuries, particularly fractures of the scapula (including the body, neck, coracoid process, and glenoid) and clavicle. It is also valuable for evaluating triple disruptions involving multiple SSSC structures, mapping fracture patterns, and assessing alignment. Intra-articular fractures of the glenoid, which are difficult to visualize on X-rays, can be clearly delineated using CT, making it essential for pre-operative planning in surgical cases.^29^ 3- or 4-dimensional CT helps to visualize the SSSC ring structure both statically and dynamically, whether disrupted or not.^19^ Elderly or osteoporotic patients may also sustain complex disruption patterns after relatively low-energy mechanisms, supporting a lower threshold for CT-based evaluation when symptoms exceed plain radiographic findings.

On the other hand, MRI is the modality of choice for soft tissue evaluation and identifying associated ligamentous injuries. It is particularly effective in detecting injuries to the coracoacromial, CC, and AC ligaments, as well as rotator cuff tears and surrounding soft tissue damage, such as labral tears, anterior labroligamentous periosteal sleeve avulsion lesions, and muscle tears or avulsions.^2^ MRI is also useful in evaluating SC joint instability and detecting bone marrow edema or subtle fractures that may not be apparent on X-rays or CT, especially in low-energy trauma cases. While CT excels in precise bony reconstruction, MRI complements it by providing detailed insights into ligamentous, cartilaginous, and soft tissue injuries, ensuring comprehensive assessment and management of SSSC injuries.^16^

In addition to imaging modality selection, certain quantitative radiological parameters are also crucial in guiding surgical decision-making for SSSC injuries. Specifically, glenopolar angle, angulation, and medial and/or lateral displacement serve as key indicators of instability and the need for operative intervention. A glenopolar angle of less than 30° has been associated with poor functional outcomes and is considered a relative indication for surgical correction, especially in displaced scapular fractures. Similarly, significant angulation or translational displacement, particularly in the coronal and axial planes, may suggest mechanical instability of the shoulder girdle and impaired biomechanics of the scapulothoracic articulation. These parameters assist in identifying cases where anatomical reduction cannot be achieved conservatively and help in surgical planning, especially in complex or triple-disruption injuries involving multiple SSSC elements.^4^^,^^10^^,^^30^

## Treatment strategies

### Single disruptions

**Figure 2:**
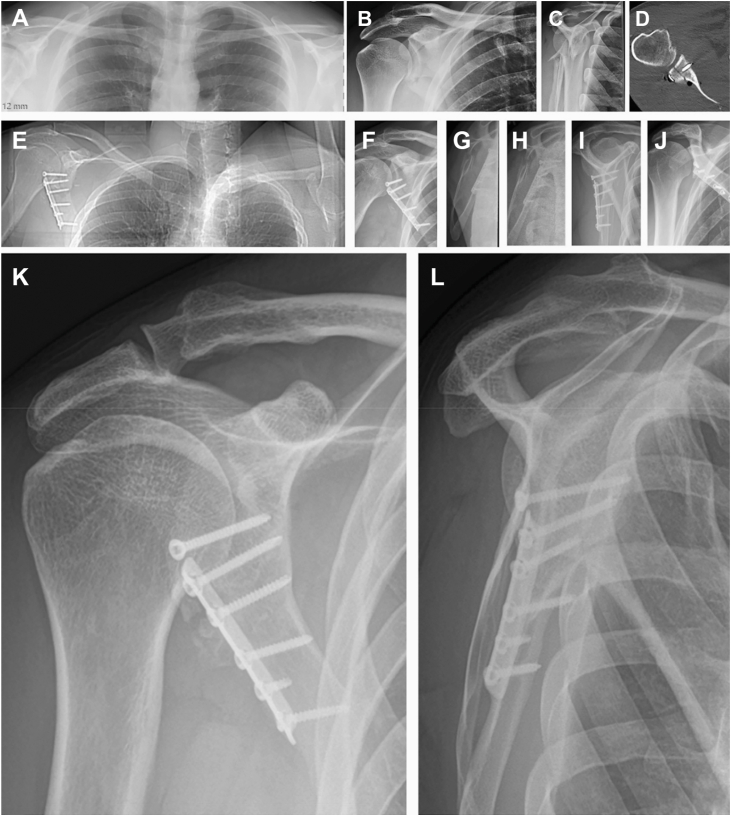
Single disruption injury of SSSC and its treatment. A right scapular fracture involving the glenoid cavity and scapular neck occurred in a 42-year-old patient following a motorcycle accident. (**A**) Panoramic radiograph of the clavicle. (**B**) Anteroposterior radiograph of the right shoulder. (**C**) Scapular Y-view radiograph of the right shoulder. (**D**) Axial CT image at the level of the glenohumeral joint demonstrating a scapular shaft fracture extending into the superior portion of the glenoid cavity. (**D–H**) Immediate post-operative radiographs (axial CT in D) following open reduction and internal fixation using a 5-hole one-third tubular plate and a free 3.5 mm cortical screw. (**I** and **J**) Radiographs at 4-month follow-up, showing stable fixation and ongoing fracture healing. (**K** and **L**) Radiographs at 4-year follow-up, demonstrating full fracture union and maintained alignment without implant-related complications. Although single disruptions are usually managed conservatively, the presence of glenoid involvement and the potential for articular or mechanical compromise justified operative fixation in this case. Open reduction and internal fixation using a 5-hole one-third tubular plate and a free 3.5-mm cortical screw achieved complete fracture union and maintained alignment at 4-year follow-up without implant-related complications. Therefore, the key treatment question in single disruptions is not simply whether one structure is injured, but whether that injury preserves or compromises the functional stability of the shoulder girdle. *SSSC*, superior shoulder suspensory complex; *CT*, computed tomography.

Single disruptions of the SSSC usually preserve ring stability and are therefore commonly suitable for nonoperative management. Nondisplaced or minimally displaced isolated coracoid fractures,^13^^,^^53^ low-grade ACJ injuries,^18^ and many isolated scapular fractures^15^^,^^41^^,^^45^ can be treated with sling immobilization, pain control, and early progressive shoulder mobilization. This approach is supported by the general principle that most scapular fractures heal uneventfully without surgery when displacement is limited and the SSSC ring remains mechanically intact.^20^ However, “single disruption” should not automatically be equated with “benign injury.” Surgical treatment should be considered when the isolated lesion is substantially displaced, intra-articular, associated with glenoid involvement, produces mechanical impingement, compromises the superior or inferior strut, or prevents early functional rehabilitation.^20^ The recent literature on high-grade AC injuries further supports an individualized approach rather than a uniform operative strategy. Contemporary comparative evidence suggests that selected high-grade ACJ injuries may achieve satisfactory clinical outcomes with nonoperative care, although surgery may improve radiographic reduction in some cases.^34^ In a recent cohort of 48 patients with acute Rockwood type-5 ACJ dislocations, 15% of patients initially treated nonoperatively eventually required surgery; however, patients who achieved successful nonoperative treatment demonstrated clinical outcomes comparable to those treated with initial operative stabilization.^3^ These findings suggest that even high-grade ACJ disruption, when occurring as an isolated single-point SSSC injury, should not automatically be regarded as an absolute indication for surgery. Instead, treatment should be individualized according to pain, functional demand, horizontal and vertical instability, reducibility, radiographic alignment, and the patient's ability to comply with follow-up. Conversely, acute or chronic ACJ dislocations, lateral clavicle fractures with CC ligament disruption, and revision cases may require reconstructive strategies when persistent instability or functional impairment is expected. In this context, Ingoe et al^25^ described the Queensland Unit for Advanced Shoulder Research (QUASR) 3-Tunnel Technique, which uses a Ligament Augmentation and Reconstruction System (LARS; Surgical Implants and Devices, Arc-sur-Tille, France) artificial ligament to reconstruct the SSSC in patients with acute, chronic, and revision ACJ dislocations as well as lateral clavicle fractures, reporting favorable midterm outcomes in a cohort of 26 patients without tunnel fractures. This technique does not imply that all ACJ injuries require surgery, but it illustrates how modern reconstruction can be used when the single disruption behaves as a clinically unstable lesion.^25^ Our institutional illustrative case of a single SSSC disruption involving the glenoid cavity and scapular neck demonstrates this principle (Fig. 2).

### Double disruptions

Double disruptions represent the critical threshold at which the SSSC ring may become mechanically unstable. Because a ring-like structure may tolerate one disruption more easily than 2, the coexistence of 2 fractures, 2 ligamentous injuries, or 1 fracture with one ligamentous injury can convert an otherwise stable shoulder girdle injury into an unstable lesion. Before the ring concept was emphasized by Goss, these injuries were often evaluated as isolated fractures or dislocations, which could lead to underestimation of the combined biomechanical instability.^21^ Inadequate recognition or insufficient stabilization of an unstable double disruption may result in delayed union, nonunion, malunion, persistent pain, and long-term functional impairment.^18^ However, high-quality comparative studies evaluating long-term outcomes of operative versus nonoperative treatment remain limited. Therefore, treatment should be individualized according to fracture displacement, glenopolar alignment, involvement of the glenoid or scapular neck, AC/CC stability, patient physiology, bone quality, and functional demand. Historically, clavicular osteosynthesis alone has been recommended in selected floating shoulder patterns because restoration of clavicular length and alignment may indirectly improve scapular position and reduce deforming forces across the scapular neck. Herscovici et al^24^ and Low et al^33^ advocated clavicle fixation in double disruptions of the SSSC to reduce the risk of scapular neck malunion. Similarly, Rikli et al^46^ emphasized that clavicle fractures or AC/SC joint disruptions can destabilize the shoulder girdle and that clavicle stabilization alone may indirectly reduce associated scapular fractures in selected cases. However, fixation of the clavicle alone may be insufficient when there is marked scapular neck displacement, glenoid involvement, acromial or coracoid displacement, persistent AC/CC instability, or failure of indirect reduction. Therefore, direct fixation of the scapula, coracoid process, acromion, or ACJ should be considered when CT demonstrates persistent malalignment or when intraoperative assessment confirms residual instability after fixation of the dominant lesion. The available clinical literature supports this stability-based approach. Oh et al^41^ reported that surgical treatment of double SSSC disruptions provided better functional outcomes, facilitated early rehabilitation, and reduced the risk of scapular malunion compared with conservative treatment. In the series by Tuor et al^52^, surgical reconstruction of double SSSC injuries resulted in bone union in all patients and allowed return to preinjury activity levels. Westphal et al^54^ also reported favorable outcomes after operative treatment of double SSSC disruptions; in their series, all coracoid fractures were Ogawa type 1, 5 of 6 acromion fractures were Kuhn type 3, and at least one fracture site was stabilized in each case. Taken together, these studies suggest that the key principle is not mandatory fixation of every injured structure, but restoration of the mechanically dominant component or components required to re-establish SSSC stability.

Double disruptions involving ACJ dislocation and coracoid process fracture require particular attention because the coracoid functions as a key osseoligamentous link between the clavicle and scapula through the CC and CA ligament complexes. In this injury pattern, stabilization may be achieved by fixation of the coracoid process alone, ACJ stabilization alone if the coracoid fracture reduces indirectly and remains stable, or combined fixation of both components.^43^ Coracoid fixation can be performed using cannulated screws, whereas ACJ stabilization may be achieved vertically with screws or horizontally with a hook plate, which is generally removed after fracture healing, commonly within 3–4 months.^35^ Conversely, CC screws and suture-button constructs have been discouraged in the setting of coracoid fractures, and transarticular wires carry risks of migration and breakage.^35^ Recent literature continues to support this individualized principle. Dimitriu et al^12^ described an unusual pattern of ACJ instability with a coracoid base fracture and achieved good early radiographic and clinical outcomes using hook plate stabilization of the ACJ combined with screw fixation of the coracoid. This recent report reinforces that combined fixation can be appropriate when both the ACJ and coracoid fracture contribute to mechanical instability.

**Figure 3:**
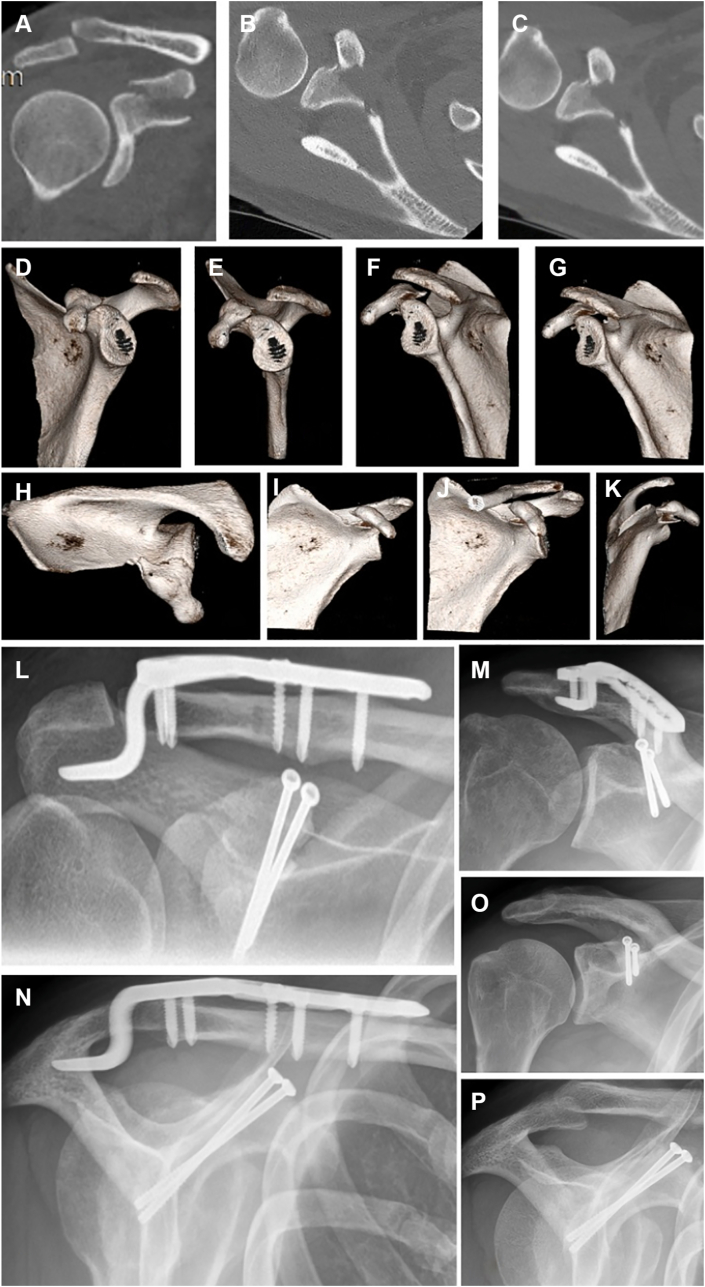
Double disruption injury of SSSC and treatment with hook plate and screws. A fracture of the coracoid process with acromioclavicular joint separation of the right shoulder occurred in a 32-year-old male treated with open reduction of the AC joint and implantation of a hook plate, along with closed reduction of the coracoid process and fixation using 2 screws; the hook plate was removed after 3 months. (**A–K**) 2- and 3-dimensional CT images demonstrating a double disruption of the SSSC of the right shoulder, including a fracture of the coracoid process with ACJ separation of the right shoulder. (**L–N**) Immediate post-operative radiograph (L) and post-operative radiographs obtained 3 months after open reduction of the ACJ and implantation of a 15-mm 6-hole hook plate, along with closed reduction of the coracoid process and fixation using 2 3.5 mm screws. (**O–P**) Radiographs at 3-month follow-up showing complete fracture union, proper alignment, and absence of implant-related complications following removal of the hook plate. In the case of coracoid process fracture combined with ACJ separation, fixation of both the ACJ and coracoid process was selected to restore the clavicle–scapula linkage and prevent persistent ring instability. Open reduction of the ACJ with implantation of a 15-mm 6-hole hook plate, together with closed reduction of the coracoid process and fixation using 2 3.5-mm screws, achieved complete fracture union, proper alignment, and no implant-related complications at 3-month follow-up after hook plate removal. *SSSC*, superior shoulder suspensory complex; *ACJ*, acromioclavicular joint; *CT*, computed tomography.

**Figure 4:**
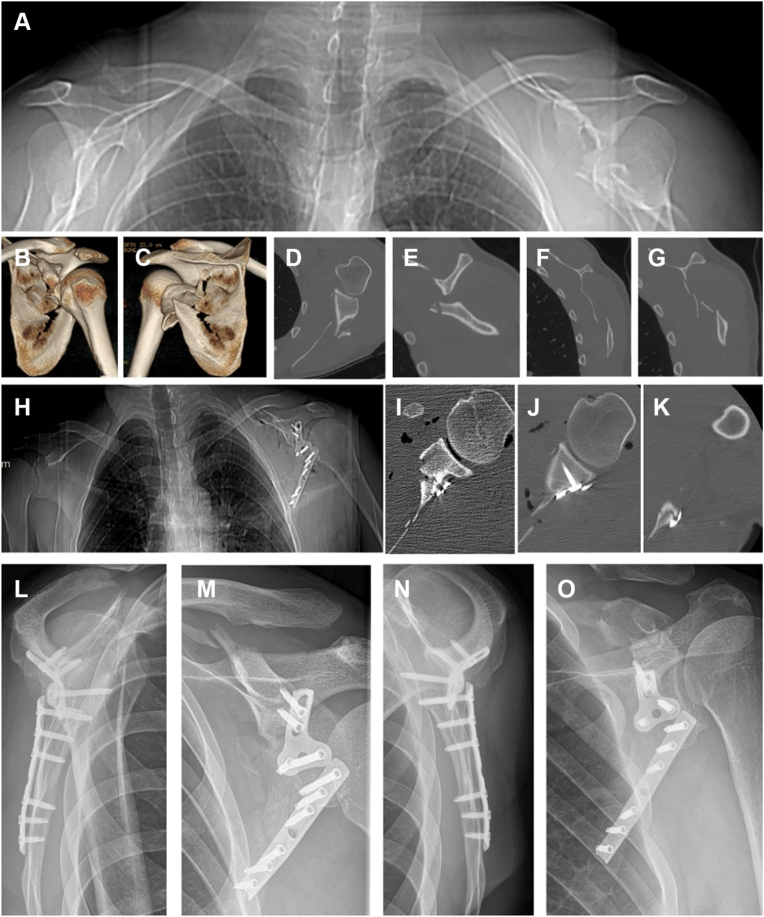
Double disruption injury of SSSC and its treatment. The injury occurred in a 29-year-old patient as a result of a sports-related trauma during a skiing accident. (**A**) Severely displaced scapular fracture involving the inferior displacement of the scapular body and coracoid process. (**B** and **C**) 3-dimensional CT image of the fracture. (**D**-**G**) Axial and coronal CT sections of the fracture. (**H-K**) Open reduction and internal fixation of the scapula using plate osteosynthesis. (**L-M**) Radiographs at 2-month follow-up, showing stable fixation and ongoing fracture healing. (**N-O**) Radiographs at 8-year follow-up, demonstrating full fracture union and maintained alignment without implant-related complications. A severely displaced scapular fracture with inferior displacement of the scapular body and coracoid process was treated with open reduction and internal fixation using plate osteosynthesis. Complete fracture union and maintained alignment were observed at 8-year follow-up without implant-related complications. These cases emphasize that double disruptions should be treated according to the unstable component of the ring rather than by a single universal fixation rule. Overall, the practical treatment principle for double disruptions is selective restoration of stability. Fixation of one dominant lesion may be sufficient when reduction of the remaining components is achieved indirectly and the SSSC ring becomes stable. Conversely, combined fixation should be considered when displacement persists, when the coracoid or acromion remains unstable, when glenoid or scapular neck involvement threatens shoulder mechanics, or when AC/CC instability prevents restoration of the clavicle–scapula relationship. Thus, the decision is not simply “operative versus nonoperative,” but rather whether the injured ring can maintain alignment and permit early functional rehabilitation without progressive deformity or chronic instability. *AC*, acromioclavicular; *CT*, computed tomography; *CC*, coracoclavicular; *SSSC*, superior shoulder suspensory complex.

Newer complex double-disruption patterns also highlight that intra-articular glenohumeral stability must not be overlooked. Cruz et al^11^ reported a rare anterior glenohumeral fracture-dislocation with critical bony Bankart involvement combined with acromion and coracoid fractures, representing a double SSSC disruption associated with major intra-articular instability. In that setting, reconstruction of the glenoid fossa and fixation of the acromion and coracoid were required to restore both glenohumeral joint stability and SSSC integrity.^11^ This case illustrates that double disruptions are not a homogeneous entity: some primarily involve the clavicle–scapula ring, whereas others combine SSSC instability with glenohumeral articular instability.^11^ Therefore, careful CT-based evaluation of the glenoid, scapular neck, coracoid, acromion, and AC/CC complex is essential before deciding whether one-site or multisite fixation is required. Our institutional double-disruption cases further illustrate this treatment logic (Figs. 3 and 4).

### Triple disruptions

Triple disruptions of the SSSC are rare injuries involving 3 distinct failures of the shoulder girdle ring and should generally be considered mechanically unstable until proven otherwise.^5^^,^^21^^,^^28^^,^^38^ Although they are classically associated with high-energy trauma, the key treatment implication is mechanical rather than purely etiologic: CT-based assessment is required to define all disrupted components, determine which structures are responsible for instability, and plan selective stabilization, particularly when clinical symptoms exceed radiographic findings.^5^^,^^28^^,^^36^^,^^54^

The available evidence on triple disruptions is dominated by case reports and small case series, which limits the possibility of strong comparative recommendations. Nevertheless, a consistent treatment principle emerges from the literature: the goal is not necessarily to fix every injured structure but to restore the mechanically dominant unstable components required to re-establish SSSC continuity, scapulothoracic mechanics, glenohumeral alignment, and early functional rehabilitation. If left untreated or insufficiently stabilized, triple disruptions may lead to delayed union, nonunion, malunion, chronic instability, persistent pain, subacromial or scapulothoracic impingement, and impaired shoulder function.^5^^,^^21^^,^^28^^,^^38^ Therefore, early recognition and CT-based characterization of all disrupted components are essential for avoiding underdiagnosis and undertreatment. Westphal et al^54^ provided an important example of how additional disruptions can convert an apparently double injury into a more complex unstable pattern. In their surgical series, one patient had a lateral clavicle fracture in addition to a double disruption, representing a third SSSC disruption, whereas another had a medial clavicle strut fracture; both required stabilization at 2 sites. Despite the rarity of these patterns and the presence of concomitant injuries, surgical stabilization of at least one fracture site led to satisfactory functional outcomes.^54^ This supports the concept that treatment should be based on restoration of ring stability rather than on a rigid requirement to fix all injured sites.

Published triple-disruption cases further demonstrate that selective stabilization can be sufficient when the dominant unstable components are addressed. Lecoq et al^32^ reported a triple SSSC fracture involving the coracoid, acromion, and clavicle, in which only the displaced coracoid fracture was treated surgically with open reduction and internal fixation, despite associated displacement of the coracoid and acromion. Barrera et al^5^ described a triple disruption involving a Kuhn type-2 acromial fracture, ACJ separation, and Ogawa type-1 coracoid fracture; the acromion was fixed with a reconstruction plate, and the ACJ was stabilized with a distal clavicle hook plate, whereas the coracoid was not fixed because it was anatomically reduced and the SSSC was stable after fixation of the other components.^5^ The patient achieved union and full active range of motion at 1-year follow-up. Similarly, Tamimi et al^49^ reported a triple SSSC injury involving a Neer type-2 clavicle fracture, Kuhn type-3 acromion fracture, and Ogawa type-1 coracoid fracture; the clavicle was stabilized with Kirschner wires, the acromion with cannulated screws, and the coracoid was managed conservatively, resulting in excellent function at 24 months, with a Constant Murley score of 90 points and a Quick Disabilities of the Arm, Shoulder and Hand (QuickDASH) score of 2.3 points. These cases show that a minimally displaced or indirectly reduced coracoid fracture may occasionally be treated nonoperatively when fixation of the dominant unstable lesions restores ring stability. In contrast, other reports support multisite fixation when several components contribute to persistent instability or malalignment. Kim et al^28^ reported a triple SSSC disruption associated with an ipsilateral humeral shaft fracture; the coracoid fracture was fixed with a cancellous screw, the acromial fracture with tension band wiring, the ACJ with 3 transarticular Steinmann pins, and the humeral shaft fracture with an intramedullary nail. At 5-year follow-up, the patient had pain-free full range of motion and was able to perform daily activities without limitation.^28^ Jung et al^27^ treated a triple disruption involving fractures of the coracoid process, acromion, and distal clavicle using Kirschner wires and cannulated screws, achieving radiographic union, full active shoulder range of motion, absence of pain or neurologic symptoms, and no instability or complications. Wu et al^55^ described a triple disruption involving fractures of the coracoid, acromion, and distal clavicle; the acromion was fixed with a 3.5-mm reconstruction locking plate, the distal clavicle with a hook plate, and the coracoid with a 4.0-mm cannulated titanium screw, resulting in excellent functional outcomes. These reports suggest that multi-site fixation is appropriate when multiple displaced structures collectively maintain the instability of the SSSC ring.

Triple disruptions may also involve less common combinations, including scapular spine or ACJ lesions. Gonçalves et al^18^ presented a case involving coracoid base fracture, ACJ dislocation, and scapular spine fracture; the scapular spine was fixed with a 3.5-mm plate and screws, the ACJ with a 2.5-mm Steinmann pin, and the coracoid with a 3.5-mm cannulated screw, leading to full union and painless full shoulder motion within 3 months. Jaën et al^26^ reported a triple disruption consisting of a Neer type-1 distal clavicle fracture, displaced acromion fracture, and Ogawa type-1 coracoid process fracture.^26^ Fixation included multiple plates for the clavicle and acromion, together with a transglenoid endobutton and cortical screws for coracoid stabilization. After hardware removal and secondary osteosynthesis of the coracoid process, the patient had good shoulder function and no pain 3 months after the second surgery; by 6 months, active motion included 160° of flexion, 150° of abduction, 50° of external rotation, and internal rotation to the L2 level, without limitation in daily activities.^26^

**Figure 5:**
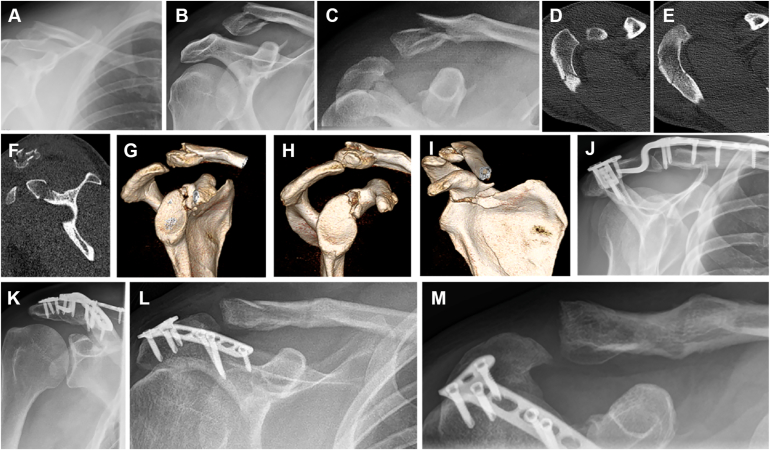
Triple disruption injury of SSSC and its treatment. The injury occurred in a 38-year-old patient following a high-energy bicycle accident. (**A–I**) Initial radiographic, 2- and 3-dimensional CT images demonstrating a triple disruption of the SSSC of the right shoulder, including fractures of the lateral clavicle, acromion, and the base of the coracoid process. (**J** and **K**) Post-operative radiographs obtained 2 months after open reduction and internal fixation, prior to implant removal. Fixation included a 3.5-mm T-shaped locking compression plate (LCP-plate) for the acromion and a hook plate for the lateral clavicle fracture. (**L** and **M**) Radiographs at 6-month follow-up showing complete fracture union, proper alignment, and absence of implant-related complications following removal of the hook plate 4 months before. *SSSC*, superior shoulder suspensory complex; *CT*, computed tomography. A triple disruption of the right SSSC involving fractures of the lateral clavicle, acromion, and base of the coracoid process occurred in a 38-year-old patient after a high-energy bicycle accident. Open reduction and internal fixation were performed using a 3.5-mm T-shaped locking compression plate for the acromion and a hook plate for the lateral clavicle. This strategy restored the dominant unstable components of the SSSC ring and achieved complete fracture union, proper alignment, and no implant-related complications at 6-month follow-up after hook plate removal. The case also reinforces the importance of CT-based pre-operative planning, because the true number and configuration of disruptions may be underestimated on plain radiographs. Overall, the treatment principle for triple disruptions is selective but decisive stabilization. Displaced acromion, clavicle, scapular spine, glenoid neck, and coracoid fractures should be addressed surgically when they contribute to instability, malalignment, impingement, loss of the deltoid lever arm, or inability to begin rehabilitation. Conversely, a minimally displaced or anatomically reduced component may be managed nonoperatively if fixation of the dominant unstable structures restores ring stability and intraoperative or radiographic assessment confirms acceptable alignment. Thus, the decision should not be based solely on the number of disrupted structures, but on which components are mechanically responsible for persistent instability. The treatment principle for quadruple disruptions is individualized restoration of functionally relevant stability. In young, active, high-demand, or reconstructable patients, early operative fixation of the dominant unstable components should be strongly considered to restore SSSC continuity, glenohumeral congruity, scapulothoracic mechanics, and the ability to begin rehabilitation. In elderly, low-demand, medically complex, or osteoporotic patients, nonoperative treatment may be reasonable when alignment is acceptable, pain is manageable, neurovascular status is intact, and the expected morbidity of extensive surgery outweighs the likely functional benefit. Therefore, the central decision is not simply whether 4 structures are disrupted, but whether the remaining shoulder girdle can maintain alignment, permit functional recovery, and avoid chronic instability without operative reconstruction. *CT*, computed tomography; *SSSC*, superior shoulder suspensory complex.

Associated injuries should be actively sought because they may influence surgical timing, patient positioning, fixation sequence, and rehabilitation. Mulawka et al^36^ reported that patients with triple SSSC disruptions often had associated injuries, particularly spinal and peripheral nerve lesions. Although some residual reduction in shoulder strength was observed, open reduction and internal fixation of the scapula resulted in satisfactory functional outcomes.^36^ These observations reinforce that triple disruptions should be evaluated not only as shoulder injuries but also as potential markers of high-energy trauma and systemic injury burden. Our institutional triple-disruption case further illustrates the principle of selective restoration of stability (Fig. 5).

### Quadruple disruptions

Quadruple disruptions represent the most complex end of the SSSC injury spectrum. These injuries are exceedingly rare and usually reflect severe disruption of both the osseoligamentous ring and the stabilizing struts of the shoulder girdle. They are often associated with polytrauma, high-energy lateral shoulder impact, glenohumeral fracture-dislocation, thoracic trauma, rib fractures, spinal injury, peripheral nerve injury, or ipsilateral upper-extremity fractures.^36^^,^^44^ Because 4 components of the ring are disrupted, the risk of persistent instability, malunion, nonunion, chronic pain, impaired scapulothoracic mechanics, and delayed functional recovery is substantial if the injury is underestimated or undertreated. However, quadruple disruption should not be considered a single uniform treatment category. Management should be individualized according to patient age, physiological reserve, bone quality, activity level, occupational or athletic demand, reconstructability of the fragments, associated injuries, and realistic rehabilitation goals. In young, active, or high-demand patients, the available literature generally supports timely surgical stabilization of the dominant displaced components when acceptable alignment and stability cannot be reliably achieved nonoperatively. Sarwari et al^47^ recently reported a 34-year-old professional cyclist who sustained a quadruple SSSC disruption after high-energy trauma, involving fractures of the distal clavicle, acromion, coracoid, and glenoid, together with an olecranon fracture. Because nonoperative management was considered likely to result in persistent instability, the distal clavicle, acromion, and coracoid were surgically fixed, together with olecranon stabilization.^47^ The patient regained full function within 3 months and returned to competitive cycling, supporting the concept that high-demand patients with reconstructable quadruple disruptions may benefit from early operative restoration of SSSC integrity.^47^ Quirarte et al^44^ similarly reported a complex quadruple disruption pattern associated with Rockwood type 5 ACJ separation, fractures of the glenoid, coracoid base, scapular body, and proximal humerus, and posterior shoulder dislocation. Surgical management required a combined reconstructive strategy, including fixation with a proximal humerus locking plate, lag screws for the glenoid, an L-shaped plate for the glenoid neck, and a distal clavicle plate for the scapular body; ACJ stabilization was achieved using a hook plate, which also contributed to coracoid stabilization.^44^ Function improved progressively after surgery: at 2 months, active abduction and forward flexion were 0°–40°, external rotation was 20°, and internal rotation reached L3; after hook plate removal at 7 months, forward flexion improved to 85°, external rotation to 10°, and internal rotation to the scapular spine.^44^ At nearly 3 years post-operatively, the patient achieved an ASES score of 98 with near-full range of motion and returned to work-related activities as an electrician.^44^ This case illustrates that even highly complex quadruple disruptions may recover well when the reconstructive strategy restores the main structural components required for glenohumeral and scapulothoracic function. The series by Mulawka et al^36^ further emphasizes that quadruple SSSC disruptions are frequently associated with systemic injury burden, including spinal and peripheral nerve lesions. Although some reduction in shoulder strength may persist, open reduction and internal fixation of the scapular component can result in satisfactory functional outcomes.^36^ This observation is clinically important because treatment planning in quadruple disruptions should not focus only on radiographic reduction of the shoulder girdle. Associated neurologic, thoracic, spinal, and upper-extremity injuries may influence timing of surgery, surgical positioning, fixation sequence, post-operative rehabilitation, and final functional recovery. Nevertheless, surgery is not mandatory in every quadruple disruption. Toft et al^50^ presented a quadruple SSSC disruption involving fractures of the glenoid, coracoid, and acromion, together with ACJ dislocation, that was treated conservatively. At 1-year follow-up, the patient achieved acceptable functional outcomes, and the authors emphasized that nonoperative treatment may be a viable option in selected elderly, low-demand patients with relevant comorbidities.^50^ This case is important because it prevents overgeneralization: although quadruple disruptions are mechanically severe, the risks and benefits of extensive reconstruction must be balanced against patient frailty, bone quality, comorbidity burden, expected functional demand, and tolerance for prolonged rehabilitation.

### Complex shoulder girdle and lateral implosion patterns

**Figure 6:**
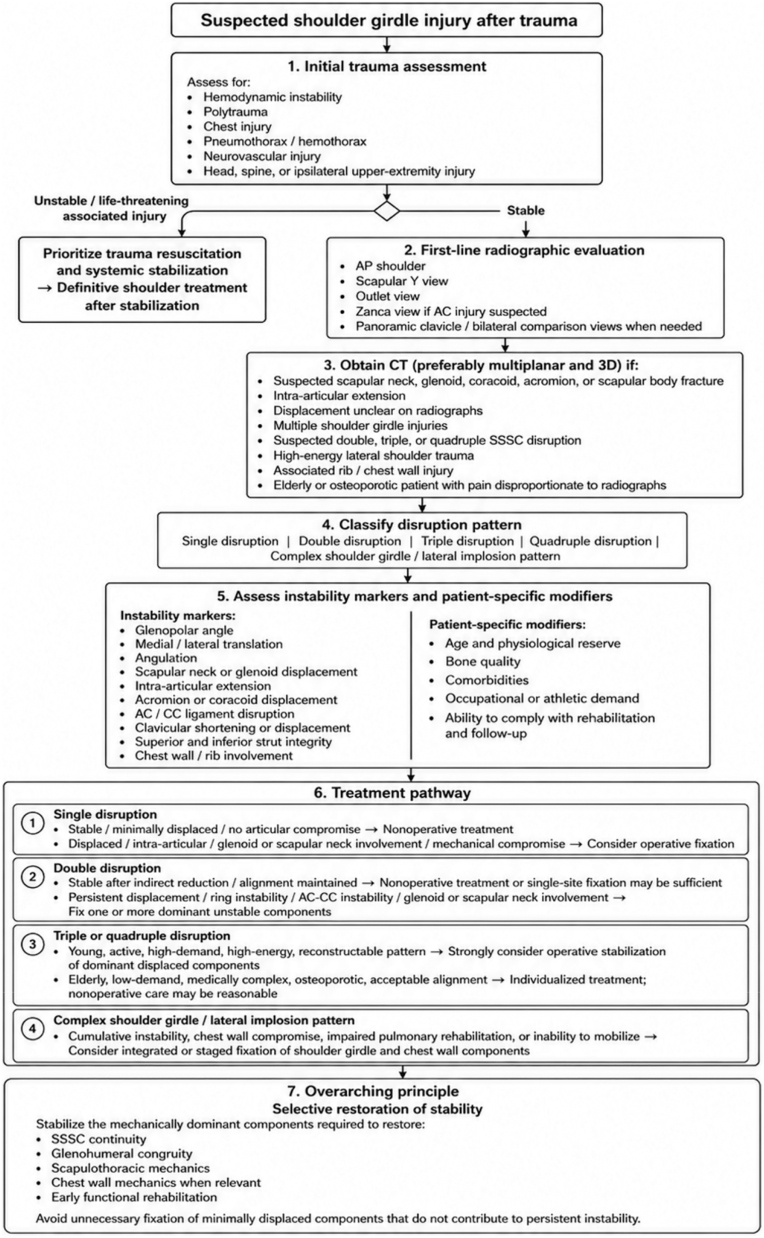
Proposed practical diagnostic and treatment algorithm for superior shoulder suspensory complex disruptions. *AC*, acromioclavicular; *AP*, anteroposterior; *CC*, coracoclavicular; *CT*, computed tomography; *SC*, sternoclavicular; *SSSC*, superior shoulder suspensory complex; *CA*, coracoacromial. The algorithm emphasizes initial trauma assessment, systematic radiographic and CT-based evaluation, classification according to the number of disrupted SSSC sites, assessment of instability markers, and individualized treatment selection.

Recent literature also suggests that SSSC disruptions should be interpreted within the broader context of shoulder girdle and chest wall mechanics. High-energy lateral shoulder trauma may produce combined injuries of the clavicle, scapula, acromion, coracoid, glenoid, and ribs that extend beyond the classic definition of single, double, triple, or quadruple SSSC disruptions. These patterns have been described as complex floating shoulder girdle injuries, scapulothoracic disruptions, or forequarter lateral implosion injuries.^8^^,^^37^^,^^51^ Although these entities are not identical to isolated SSSC disruptions, they are clinically relevant because they may involve cumulative instability of the shoulder girdle and adjacent thoracic cage. In these complex patterns, treatment decisions should account not only for SSSC ring stability but also for chest wall mechanics, pulmonary rehabilitation, deltoid function, scapulothoracic motion, glenohumeral congruity, and the feasibility of early mobilization. Chua et al^8^ recently described an integrated surgical strategy for forequarter lateral implosion injuries involving clavicle, scapula, and rib fractures, emphasizing that simultaneous osseous stabilization may restore both SSSC integrity and chest wall mechanics, thereby facilitating early shoulder mobilization and pulmonary rehabilitation. Similarly, recent reports of complex floating shoulder girdle injuries and scapulothoracic disruption treated with concurrent clavicle–scapular fixation, scapular dual-column plating, and acromion tension band osteosynthesis highlight the importance of restoring scapular architecture, deltoid attachment, and glenohumeral mechanics in rare but severe injury patterns.^37^^,^^51^ These contemporary reports do not establish high-level evidence, but they expand the clinical framework for treating complex SSSC-related injuries. They suggest that surgical thresholds may need to be adjusted when multiple adjacent structures collectively produce biomechanical instability, even if each individual fracture might otherwise be considered for nonoperative treatment. This concept is consistent with contemporary CT-based approaches to acute shoulder girdle fractures, in which multiplanar and three-dimensional assessment helps define fracture morphology, instability markers, classification, and operative planning.^14^ Thus, the concept of cumulative instability is central: the decision to operate should be based on the combined mechanical effect of all injuries rather than on any single radiographic finding. Table II summarizes the disruption patterns and corresponding treatment principles, whereas Figure 6 provides a stepwise diagnostic and treatment algorithm for clinical decision-making.

**Table II:** Practical treatment framework for superior shoulder suspensory complex disruptions according to disruption pattern, stability concept, imaging modifiers, and patient-specific treatment factors.

Pattern	Definition and stability	Key imaging and decision modifiers	Practical treatment principle	Key literature-based clinical message
Single disruption	Injury to one component of the SSSC, such as an isolated coracoid fracture, isolated scapular fracture, low-grade ACJ injury, or a single glenoid/scapular neck lesion. Ring stability is usually preserved if displacement is limited.	Standard radiographs are usually the first-line modality. CT should be considered when fracture morphology, glenoid involvement, intra-articular extension, displacement, or mechanical compromise is unclear. Important modifiers include displacement, glenoid involvement, mechanical impingement, superior/inferior strut compromise, functional demand, and ability to comply with follow-up.	Most stable, nondisplaced, or minimally displaced single disruptions can be treated nonoperatively with sling immobilization, pain control, and early progressive mobilization. Surgery should be considered when the isolated lesion is displaced, intra-articular, mechanically compromising, associated with glenoid/scapular neck involvement, or prevents early functional rehabilitation.	Most isolated scapular or minimally displaced coracoid injuries can be managed conservatively when ring stability is preserved.^13^^,^^15^^,^^20^^,^^45^^,^^53^ Selected high-grade ACJ injuries may achieve satisfactory clinical outcomes with nonoperative treatment, although some patients eventually require surgery.^3^^,^^34^ Modern reconstruction techniques, such as the QUASR 3-tunnel technique, may be useful when an apparently single ACJ/lateral clavicle disruption behaves as a clinically unstable lesion.^25^
Double disruption	Injury to 2 points of the SSSC, including 2 fractures, 2 ligamentous injuries, or one fracture combined with one ligamentous injury. This represents the critical threshold at which the ring may become mechanically unstable.	CT-based evaluation should assess fracture morphology, glenopolar alignment, scapular neck/glenoid involvement, medial or lateral translation, angulation, acromial or coracoid displacement, AC/CC stability, and whether indirect reduction occurs after fixation of the dominant lesion.	Treatment should aim to restore ring stability rather than to fix every injured structure routinely. Fixation of one dominant lesion may be sufficient when the remaining component reduces indirectly and becomes stable. Combined fixation should be considered when displacement persists, when the coracoid/acromion remains unstable, when glenoid or scapular neck involvement threatens mechanics, or when AC/CC instability prevents restoration of the clavicle–scapula relationship.	Clavicle fixation alone may indirectly improve scapular alignment in selected floating shoulder patterns.^24^^,^^33^^,^^46^ Surgical treatment of unstable double disruptions may improve function, facilitate rehabilitation, and reduce malunion risk.^41^ ACJ dislocation with coracoid fracture may require coracoid fixation, AC stabilization, or combined fixation depending on intraoperative stability.^12^^,^^35^^,^^43^ Complex double disruptions with glenohumeral fracture-dislocation or bony Bankart involvement require restoration of both glenohumeral and SSSC stability.^11^
Triple disruption	Injury to three points of the SSSC. These injuries are rare, usually high-energy, and should generally be considered mechanically unstable until proven otherwise. They may involve combinations of clavicle, acromion, coracoid, scapular spine, glenoid neck, ACJ, or ligamentous injuries.	CT is essential to define the true number and configuration of disruptions, because plain radiographs may underestimate the injury. Decision modifiers include displacement of the acromion, clavicle, coracoid, scapular spine, or glenoid neck; loss of deltoid lever arm; impingement; malalignment; associated thoracic, spinal, neurologic, or ipsilateral upper-extremity injuries; and patient age/bone quality.	Selective but decisive stabilization is recommended. The goal is not mandatory fixation of all 3 injured structures, but stabilization of the mechanically dominant components required to restore SSSC continuity, scapulothoracic mechanics, glenohumeral alignment, and early rehabilitation. Minimally displaced or anatomically reduced components may be treated nonoperatively if stability is restored after fixation of the dominant lesions.	Published triple-disruption reports show favorable outcomes after selective or multisite fixation depending on which structures maintain instability.^5^^,^^18^^,^^26^, ^27^, ^28^^,^^32^^,^^49^^,^^55^ Some cases achieved good outcomes when the coracoid was left untreated after stabilization of the dominant lesions,^5^^,^^49^ whereas others required multisite fixation of the coracoid, acromion, clavicle, or AC joint.^18^^,^^27^^,^^28^^,^^55^ Associated injuries are common and should influence surgical timing, fixation sequence, and rehabilitation.^36^^,^^54^
Quadruple disruption	Injury to 4 points of the SSSC. This represents the most complex end of the injury spectrum and is usually associated with severe osseoligamentous ring disruption, high-energy trauma, polytrauma, glenohumeral fracture-dislocation, thoracic injury, or neurologic injury.	CT should evaluate all injured ring components, glenoid involvement, scapular body/neck displacement, acromion/coracoid/clavicle fractures, AC/CC instability, and associated chest wall or upper-extremity injuries. Patient-specific modifiers are central: age, physiological reserve, bone quality, reconstructability, comorbidities, occupation, athletic demand, and expected rehabilitation tolerance.	In young, active, high-demand, or reconstructable patients, early operative fixation of the dominant unstable components should be strongly considered. In elderly, low-demand, medically complex, or osteoporotic patients, nonoperative treatment may be reasonable when alignment is acceptable, pain is manageable, neurovascular status is intact, and surgical morbidity outweighs expected functional benefit.	Recent and previous reports support early operative reconstruction in high-demand or complex quadruple disruptions, including professional athletes and patients with glenoid/proximal humerus involvement.^44^^,^^47^ However, conservative treatment may still provide acceptable outcomes in carefully selected elderly or low-demand patients.^50^ The decision should balance mechanical instability against patient-specific surgical risk and realistic functional goals.^36^^,^^44^^,^^47^^,^^50^
Complex shoulder girdle/lateral implosion patterns	Combined injuries extending beyond classic SSSC categories, including complex floating shoulder girdle injuries, scapulothoracic disruptions, or forequarter lateral implosion injuries involving the clavicle, scapula, acromion, coracoid, glenoid, ribs, and chest wall. These patterns may create cumulative instability even if individual fractures appear borderline for surgery.	3-dimensional CT is central for defining fracture morphology, cumulative instability, chest wall involvement, rib/scapular/clavicular relationships, and operative sequencing. Decision modifiers include pulmonary mechanics, chest wall stability, deltoid function, scapulothoracic motion, glenohumeral congruity, pain control, and feasibility of early mobilization.	Treatment should consider both SSSC stability and chest wall mechanics. Concurrent or staged fixation may be appropriate when combined injuries impair shoulder girdle stability, pulmonary rehabilitation, or early mobilization. The decision should be based on cumulative biomechanical instability rather than on any single radiographic finding.	Recent reports emphasize integrated surgical management of clavicle, scapula, and rib injuries to restore both SSSC integrity and chest wall mechanics.^8^ Concurrent clavicle–scapular fixation, scapular dual-column plating, and acromion tension band osteosynthesis may help restore scapular architecture, deltoid attachment, and glenohumeral mechanics in severe patterns.^37^^,^^51^ Contemporary CT-based assessment supports classification, instability evaluation, and operative planning in acute shoulder girdle fractures.^14^

*AC*, acromioclavicular; *ACJ*, acromioclavicular joint; *CC*, coracoclavicular; *CT*, computed tomography; *SSSC*, superior shoulder suspensory complex.

## Conclusion

SSSC disruptions should be evaluated as ring injuries rather than isolated fractures or ligamentous lesions. Although disruption number provides an initial estimate of instability, treatment decisions should be guided by displacement, glenopolar alignment, translational deformity, intra-articular or glenoid/scapular neck involvement, ligamentous integrity, patient physiology, bone quality, comorbidities, and functional demand. Stable single disruptions can usually be managed nonoperatively, whereas displaced, intra-articular, mechanically compromising, or function-limiting lesions may require fixation. Double disruptions represent the key threshold for potential ring instability and require CT-based assessment to determine whether nonoperative treatment, single-site fixation, or multisite fixation is appropriate. Triple-quadruple-and complex lateral implosion patterns should be interpreted according to cumulative biomechanical instability rather than mechanism or disruption number alone. This review provides a practical framework integrating radiographs, CT, selective MRI, instability markers, patient-specific modifiers, and selective restoration of stability to reduce missed instability, delayed diagnosis, and undertreatment, while acknowledging the limited evidence base.

## Disclaimers:

Funding: No funding was disclosed by the authors.

Conflicts of interest: The authors, their immediate families, and any research foundations with which they are affiliated have not received any financial payments or other benefits from any commercial entity related to the subject of this article.
